# An endonasal approach to the resection of a papillary seromucinous adenocarcinoma of the Eustacian tube

**DOI:** 10.1186/1916-0216-42-12

**Published:** 2013-02-04

**Authors:** Jordan T Glicksman, Jason H Franklin, Jessica Shepherd, Brian W Rotenberg

**Affiliations:** 1 Department of Otolaryngology, Schulich School of Medicine, University of Western Ontario, London, Ontario, Canada; 2 Department of Pathology Schulich School of Medicine, University of Western Ontario, London, Ontario, Canada; 3 St Joseph's Hospital, Department of Otolaryngology, 268 Grosvenor St, London, ON, N6A 4V2, Canada

**Keywords:** Nasopharynx, Seromucinous adenocarcinoma, Endoscopic resection, Eustacian tube

## Abstract

**Objectives:**

Papillary seromucinous adenocarcinoma of the sinonasal tract is exceedingly rare. The objectives of this case report are to describe a case of papillary seromucinous adenocarcinoma presenting in the nasopharynx and to review the literature pertaining to other similar cases.

**Methods:**

A review of the patient's chart and a review of the English literature were conducted.

**Results:**

We describe the case of a 64 year-old woman who presented with a 3-year history of epistaxis and right-sided otitis media with effusion. The patient had been followed for a known nasopharyngeal mass that had twice been biopsied and in both cases was considered a benign mass pathologically. A third biopsy was diagnosed as a low-grade papillary seromucinous adenocarcinoma. The patient was otherwise asymptomatic. The patient was referred to a multidisciplinary cancer clinic at which endoscopic resection was determined to be the preferred treatment modality. A literature review and approach to patients with nasopharyngeal masses will be presented.

**Conclusions:**

Papillary seromucinous adenocarcinoma is a rare tumor that can present in the nasopharynx. We describe the endoscopic surgical management of one such patient that presented to our care.

## Background

The nasopharynx accounts for less than 1% of malignancies in the United States. The most common malignancy in the nasopharynx is Nasopharyngeal Carcinoma (NPC), which is a form of squamous cell carcinoma (SCC) with a histopathology and behavior unique from SCC originating elsewhere in the head and neck [1]. However, the differential diagnosis for a nasopharyngeal mass remains broad. Benign lesions include but are not limited to juvenile nasal angiofibroma, thornwaldt’s cysts, papillomas, craniopharyngiomas and benign salivary gland tumors. In addition to NPC, chordomas, lymphoma, hemangiopericytoma, rhabdomyosarcroma and salivary gland tumors represent malignant lesions of the nasopharynx.

A common problem with nasopharyngeal malignancies is that patients can often present without local symptoms; rather their primary tumor will be detected during the workup of a metastasis to a cervical lymph node [2]. Tumors of the nasopharynx can present with a common set of complaints, generally due to non-specific local effects of the neoplasm. Nasal obstruction may result from mass effects and tumor bleeding can lead to epistaxis or hemoptysis. If the tumor obstructs the Eustachian tube, the patient may develop a middle ear effusion with resultant conductive hearing loss and/or tinnitus. Some patients can present with headaches, otalgia or cranial nerve deficits. The most frequently affected cranial nerves are cranial nerves III, V, VI and XII [3].

We present the case of a 64-year-old woman with a rare nasopharyngeal malignancy of salivary origin, that being papillary serous adenocarcinoma.

## Case presentation

### Patient presentation

A 64 year-old woman presented with a 3-year history of epistaxis and right-sided otitis media with effusion. She had no history of nasal obstruction, dysphagia, odynophagia, voice change, vision change, diploplia, weight loss, or B-symptoms. There was no prior history of smoking or radiation and she had minimal alcohol consumption. Prior to referral she had twice undergone biopsy of the lesion and this was first reported as a benign growth in the nasopharynx. A third biopsy was then diagnosed as a low-grade seromucinous papillary carcinoma, for which she was referred to our center for definitive management.

**Figure 1:**
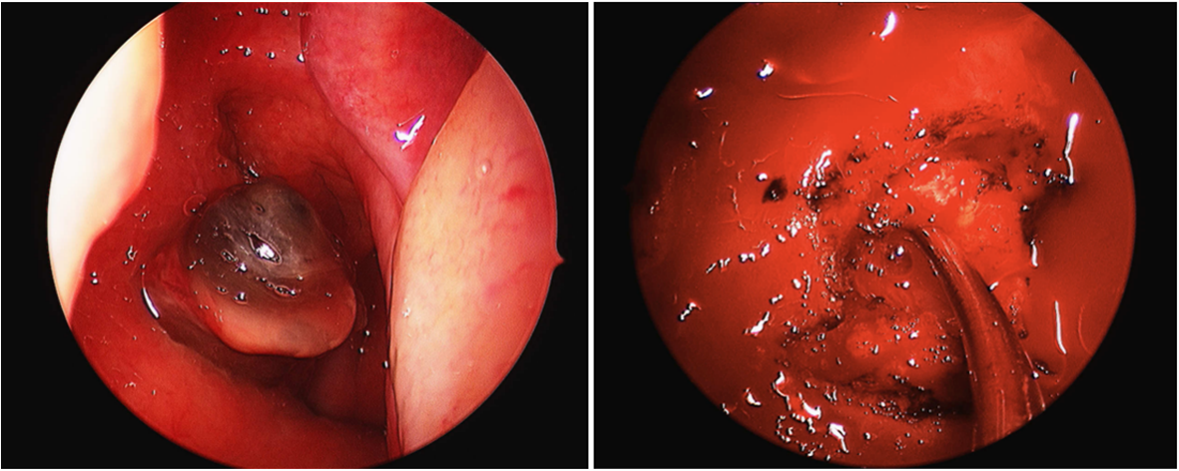
**Left: Transnasal endoscopic view of the nasopharynx demonstrating a pedunculated lesion originating from the right eustacian tube and entering the nasopharynx.** Right: Intraoperative photo of the deep aspect of the resection. The suction is pointing to the remnant of the lumen of the Eustachian tube.

Physical examination of the patient was remarkable for a right-sided tympanostomy tube and 0.5-1 cm lesion derived from the right Eustachian tube. The lesion had a fleshy papillomatous appearance. Examination of cranial nerves III-VII and IX-XII was unremarkable as was examination of the oral cavity. The patient had no palpable lymphadenopathy (Figure 1).

**Figure 2:**
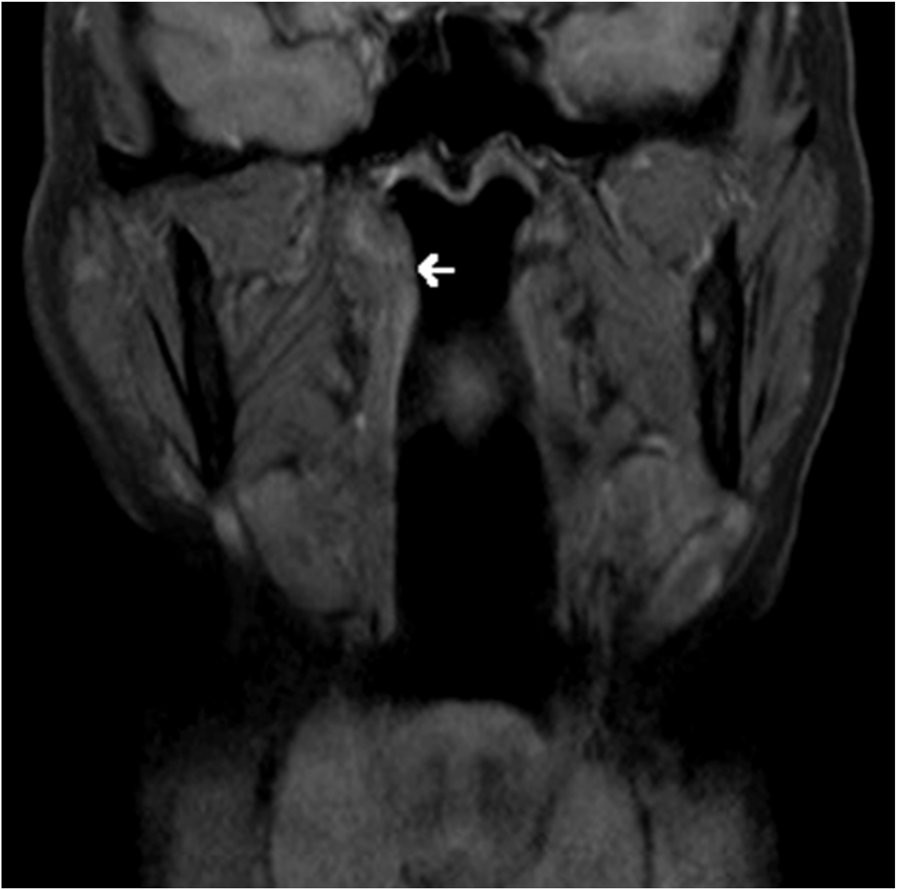
**T1**-**weighted coronal MRI image demonstrating thickening of the right Eustachian tube orifice(arrow).** The lesion appears limited to the Eustachian tube and nasopharynx, without invasion of adjacent structures.

A CT scan of the head and neck had been performed. The scan was normal in appearance with notable absence of bony erosion or involvement of adjacent structures. There was no cervical adenopathy. MRI demonstrated thickening of the right Eustachian tube orifice and confirmed that it did not involve adjacent structures such as the parapharyngeal space (Figure 2).

### Surgical technique

The patient was assessed by a Multidisciplinary Head & Neck Oncology team, wherein primary surgery via endoscopic resection was deemed the best first-line treatment. This decision was made in consideration of the small size and minimal growth of the tumor over a 3-year period, its pedunculated nature, the absence of involvement of surrounding structures and patient preference to avoid chemoradiation or more invasive open surgical approaches. For access to the lesion, a corridor was created in the nasal cavity, including development of binasal access via posterior septectomy. The tumor was visualized at its origin at the Eustachian tube orifice. The resection began with dissection of the mucosa covering the face of the sphenoid. This developed a superior plane to provide access to the tumor and expose the pterygoid wedge. The wedge was followed inferolaterally to expose the skull base, pterygoid wedge and medial pterygoid plate. The plate mucosa was stripped for an anterior tumor margin.

A linear cut was made in the posterior aspect of the torus tubarius anterior to the fossa of Rosenmuller to establish a posterior margin. The levator palatini muscle was incised inferiorly where it joined into the torus tubarius for an inferior margin. All soft tissue over the medial pterygoid plate was removed, exposing the medial pterygoid muscle, forming the lateral resection margin boundary. We then used careful cautery to form deep cuts, peeling tumor off the underlying surface of the skull base until we came to our posterior cut. The depth of dissection was approximately to the bony isthmus of the Eustachian tube. The specimen was friable and so it was removed as 2 large pieces. Frozen sections were deemed to be impractical. The surgical margins were at the level of the petrous carotid and pterygoid plates, and the tumor appeared to be completely removed at a gross level.

### Pathologic findings

**Figure 3:**
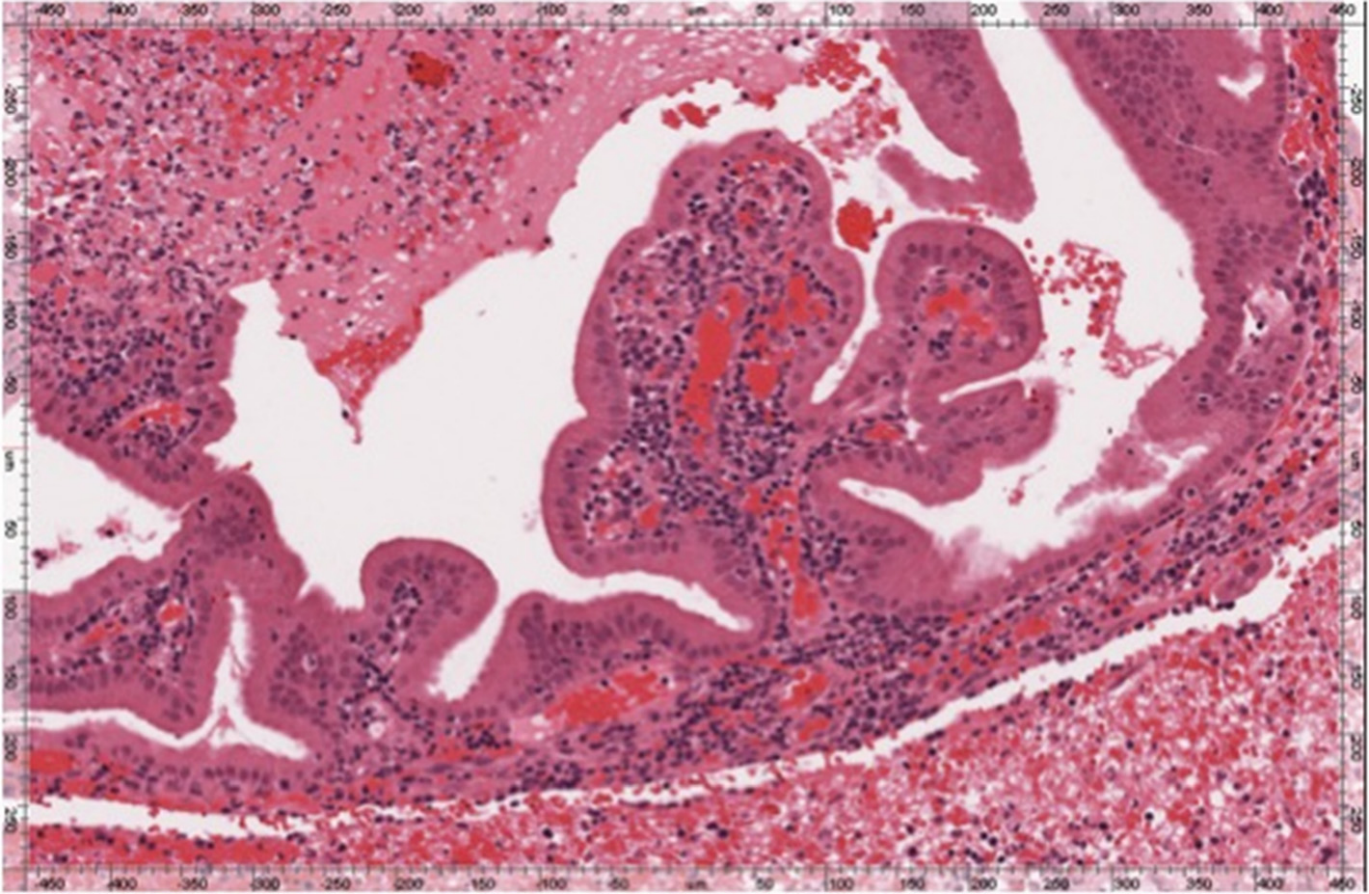
**Low power hematoxylin and eosin staining demonstrates well differentiated papillary epithelial fragments, embedded in fibrinous hemorrhage.** Mucin was demonstrated by special staining (not shown).

Final pathology was performed on the two resected fragments. The medial fragment consisted of reactive respiratory muscosa with chronic inflammation, abundant fibrinous hemorrhage and necrosis; no viable tumor was seen. The deeper specimen contained fragments of a well-differentiated papillary neoplasm consistent with low grade papillary seromucinous adenocarcinoma, as previously diagnosed, the histology having been compared to that of the previous biopsy (Figure 3).

### Follow-up

An MRI was performed 6 months post-operatively to reevaluate the operative site. There was no evidence of a lesion in the right Eustachian tube or nasopharynx. She was seen in clinic the following week. She had no symptoms of recurrence and the nasopharyngeal mucosa and Eustachian tube orifice were unremarkable on nasopharyngoscope. There was no cervical lymphadenopathy. The patient will be followed with serial physical examination and MRI to monitor for recurrence.

## Discussion

Salivary gland tumors account for less than 5% of head and neck neoplasms and typically present in the oral cavity [4]. The likelihood of finding malignant rather than benign disease in a salivary gland tumor increases as the size of the gland of origin decreases [5]. Salivary gland carcinoma accounts for less than 0.5% of all nasopharyngeal malignancies, likely owing to the low density of salivary gland tissue in the nasopharynx. Within the nasopharynx, adenoid cystic carcinoma is the most common salivary gland tumor, followed by adenocarcinoma [6].

Papillary serous adenocarcinoma is a histologically unique form of adenocarcinoma. This tumor is considered to be derived from nasopharyngeal surface epithelium rather than from underlying minor salivary glands, based on histological appearance and immunohistochemical staining profile. It is uncommon, occurring over a wide age range with no sex predilection. The commonest site is lateral or posterior wall or roof of the nasopharynx, and it presents as a soft to gritty mass with a nodular or papillary appearance. Despite a benign histological appearance, this tumor is infiltrative and tends to recur if not completely removed.

While major salivary gland tumors have their own staging system, minor salivary gland tumors are staged based on their anatomic subsite. The AJCC Cancer Staging Manual classifies the T stage of nasopharyngeal tumors based on the presence or absence of involvement of surrounding structures such as the parapharyngeal space, skull base, paranasal sinus, cranial nerves, hypoharynx, orbit, infratemporal fossa or masticator space [7]. Like other cancers, N or nodal staging and M or metastasis staging are also important in the prognostication and treatment of these tumors. Our case presented as a T1N0M0 lesion given the absence of involvement of surrounding structures, lymph nodes or distance metastasis.

Multiple treatment modalities exist for the treatment of salivary gland tumors of the nasopharynx [8]. Generally low-grade salivary gland tumors are less radiosensitive than their high-grade counterpart [9]. The traditional therapeutic approach for patients with high-grade or unresectable (T4) disease is radiotherapy. A surgical approach is more appropriate for low-grade, low-stage adenocarcinomas. While the 5-year disease survival of patients with adenocarcinoma of the nasopharynx is approximately 65%, non-randomized studies demonstrate a survival benefit with the involvement of a surgical approach [10].

To our knowledge endoscopic resection of nasopharyngeal salivary gland tumors is not yet a common practice. Since the majority of patients with nasopharyngeal malignancies present with spread to cervical lymph nodes and/or the parapharyngeal space, less invasive techniques may not be considered. Primary endoscopic approaches to other nasopharyngeal neoplasms have been described with increasing frequency. Localized benign lesions such as juvenile nasal angiofibromas can be endoscopically resected, typically after angioembolization [11,12]. Furthermore, while open surgical approaches have been the traditionally favored surgical modality for local control, there are now reports of endoscopic resection to address local recurrence of nasopharyngeal carcinoma [13,14]. For other malignancies, such as localized chondroid cordoma of the nasoppharynx and skull base, some authors advocate strongly for the use of endoscopic resection as it spares the morbidity of open approaches [15,16].

There is a paucity of literature that specifically pertains to the endoscopic resection of exocrine gland neoplasms of the nasopharynx, particularly as a primary therapy modality. Al-Sheibani et al. described their 4-handed endoscopic endonasal transpterygoid nasopharyngectomy in a retrospective study of twenty patients. Most patients in this study had advanced or recurrent disease. Among other tumors, 5 adenoid cystic and 2 adenocarcinomas were described with disease-related mortality rate of 20% and 50% respectively at the time of publication [17].

The low staging of our patient’s disease factored into our decision to pursue endoscopic resection. The absence of parapharyngeal extension or invasion of adjacent structures made the approach possible. Furthermore, the pedunculated nature of the lesion facilitated resection in that it made the tumor readily distinguishable from surrounding tissue. We were confident based on imaging and our exam findings that we would not only be able to resect the tumor but too achieve sufficient normal tissue margins with an endoscopic approach. Another factor favoring this approach was the indolent nature of this tumor, progressing minimally over a 3-year period. Our patient will require close surveillance and while we are confident that our resection was adequate, if the tumor recurs it should be detectable by physical examination.

## Conclusions

This case is interesting because it represents a rare pathological variant of an uncommon nasopharyngeal tumor. Additionally, from our review of the literature, minor salivary gland tumors of the nasopharynx appear to be derived from the mucosa of the nasopharynx itself. However, in the case of our patient, the lesion in question was in fact a pedunculated lesion that appeared to originate from within the Eustachian tube. Finally, the management of patients such as ours remains controversial. Traditionally surgical resection of nasopharyngeal malignancies has been performed by an open approach, but this paradigm appears to be evolving. In this case, a minimally invasive resection was achieved with no evidence of residual disease. This was made possible by the small size of the lesion and the lack of invasion of surrounding structures.

### Ethical approval

Written informed consent was obtained from the patient for publication of this report and any accompanying images.

## Competing interests

The authors declare that they have no competing interests.

## Authors’ contributions

All authors read and approved the final manuscript.
